# Biventricular arrhythmogenic cardiomyopathy diagnosed in a young patient: A case report with literature review

**DOI:** 10.1016/j.radcr.2023.06.034

**Published:** 2023-07-04

**Authors:** Hajar El Ouartassi, Raid Faraj, Zakariae Laraichi, Rhita Ezzahraoui, Zaineb Bourouhou, Nawal Doghmi, Mohamed Cherti

**Affiliations:** a Cardiology B Department, Ibn Sina University Hospital Center, Rabat, Morocco; b Mohammed V university Rabat

**Keywords:** Arrhythmogenic cardiomyopathy, Cardiac magnetic resonance, Padua criteria

## Abstract

Arrhythmogenic cardiomyopathy is a genetic heart muscle disease that typically affects the right ventricle. However, 2 other phenotypes affecting the left ventricle were recently discovered. Here, we report the case of an 18-year-old patient with biventricular arrhythmogenic cardiomyopathy, highlighting the challenges encountered in establishing this diagnosis. Diagnostic criteria for the left-sided phenotypic variants of arrhythmogenic cardiomyopathy were only introduced in 2020 by an international expert consensus document, known as the “Padua criteria,” they are divided in 6 categories with an emphasis on morpho-functional ventricular abnormalities and structural myocardial tissue alterations to diagnose biventricular arrhythmogenic cardiomyopathy.

## Introduction

Arrhythmogenic cardiomyopathy (ACM) is a genetic heart muscle disease characterized by the replacement of the ventricular myocardium by fibrofatty tissue [1]. The classic form of this disease typically affects the right ventricle (RV), which explains its first appellation: arrhythmogenic right ventricular cardiomyopathy. However, recent insights have led to the discovery of other phenotypes of this disease that affect the left ventricle (LV).The following clinical case is of a young patient with biventricular ACM, highlighting the challenges encountered in establishing this diagnosis.

Our paper was written according to the CARE guidelines [2].

## Case presentation

**Fig. 1:**
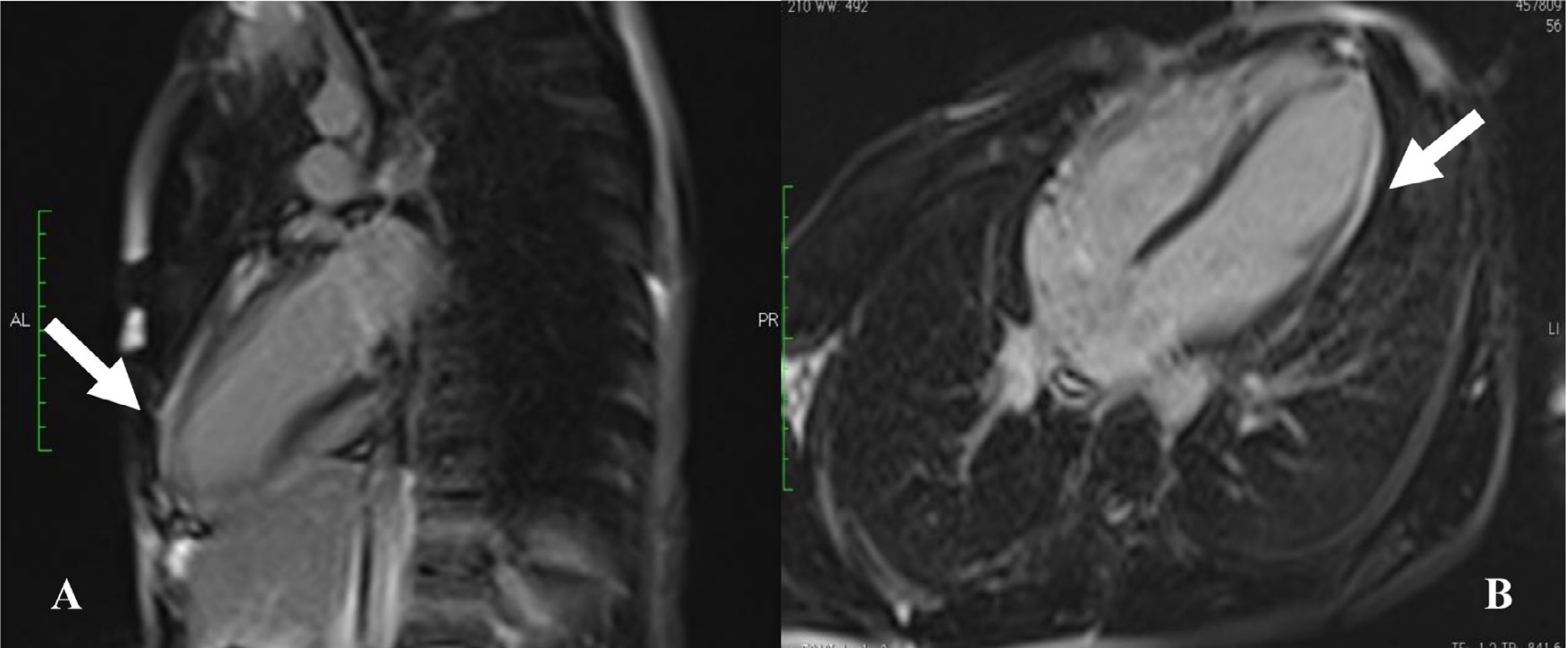
Phase sensitive inversion recuperation MRI sequences revealing late subepicardial enhancement in the form of a hypersignal after injection of gadolinium (white arrow) in the anterior and inferior wall of the left ventricle in the 2-chambers view (Image A); in the lateral wall and apical wall in the 4-chambers view (Image B).

**Fig. 2:**
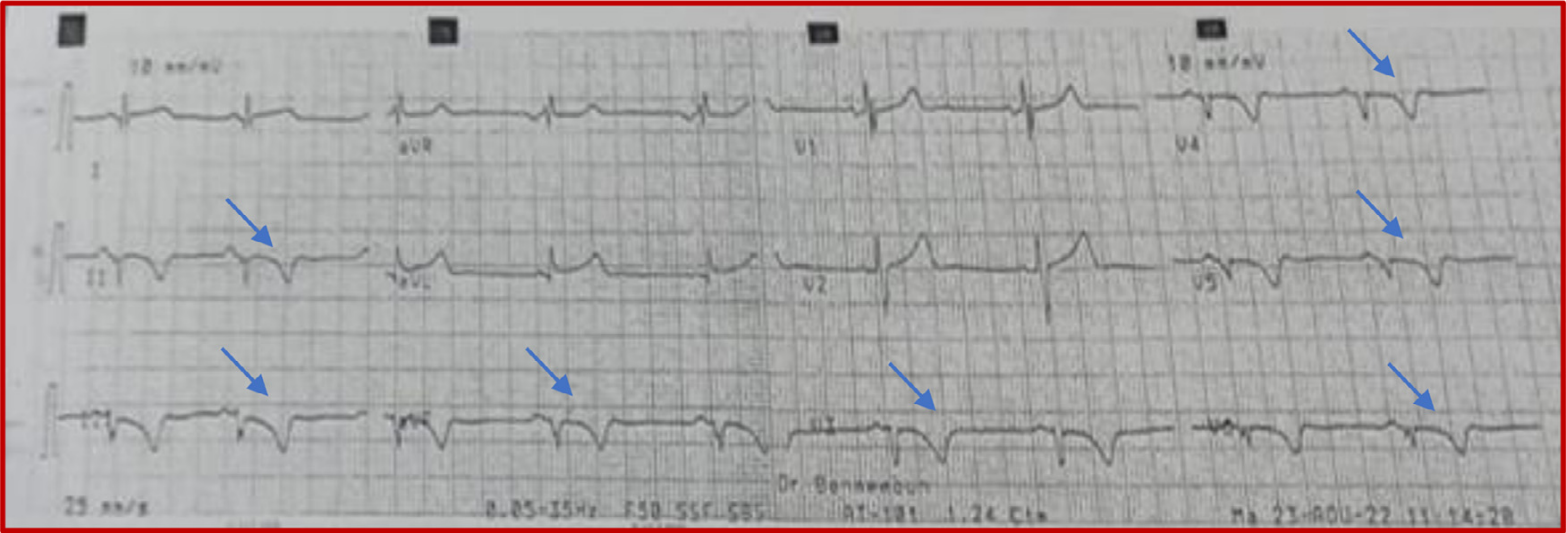
ECG revealing incomplete RBBB with repolarization disorders in inferior and left precordial leads (blue arrows).

**Fig. 3:**
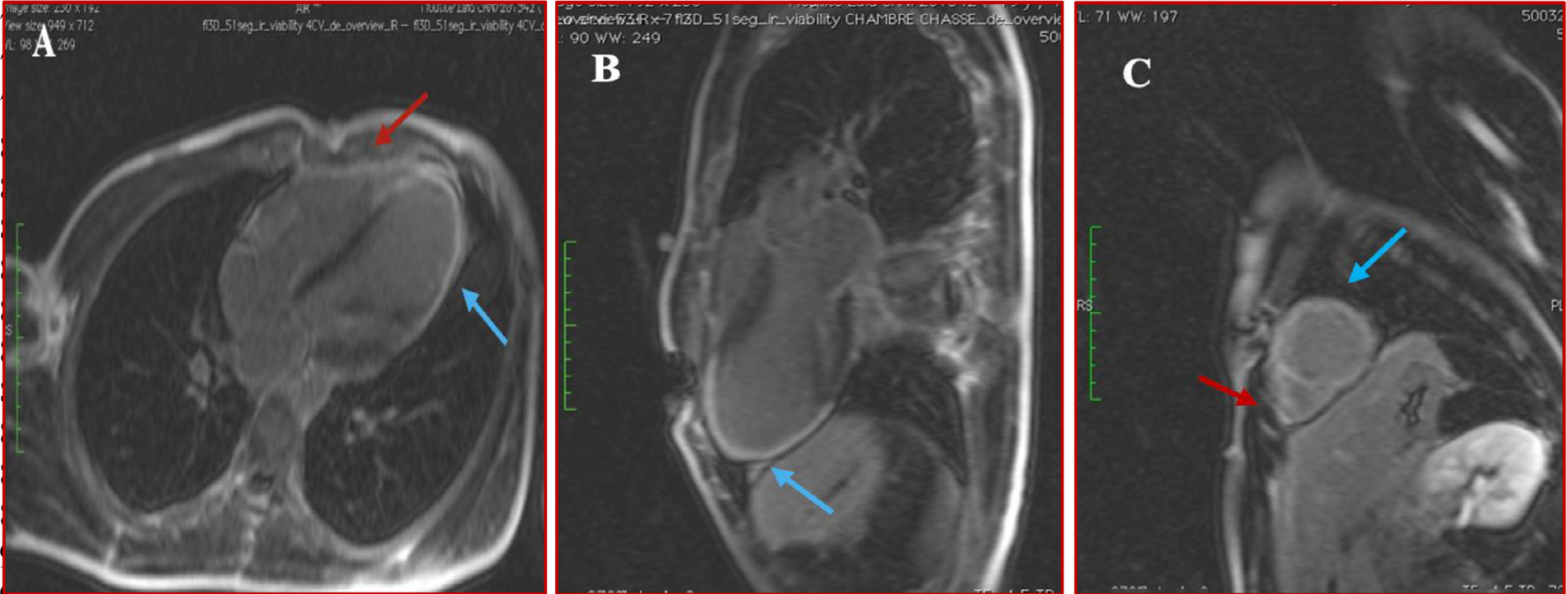
Phase sensitive inversion recuperation MRI sequences revealing progression of late gadolinium enhancement with transmural pattern (blue arrow) in lateral wall in the 4-chambers view (Image A); in the anterior and inferior wall (blue arrows) in the 2-chambers view (Image B) and in the apex in the short axis view (blue arrows) (Image C), with transmural enhancement of inferior and lateral walls of the right ventricle (red arrows).

An 18-year-old male with no personal or family medical history, presented chest pain in 2021. Physical examination was unremarkable. Electrocardiogram (ECG) revealed incomplete right bundle branch block and negative T waves in the inferolateral region. Transthoracic echocardiography (TTE) showed apical akinesia of the LV with preserved ejection fraction (EF) at 55%. Late gadolinium enhancement (LGE) imaging by cardiac magnetic resonance (CMR) identified subepicardial contrast in the apex and apical segments of the anterolateral and inferior LV walls. The RV was without abnormalities (Fig. 1). The diagnosis retained was myocarditis due the presence of foci of myocardial fibrosis. The patient was prescribed betablockers with poor compliance to the treatment. One year later, the patient presented exertional syncope (during a football match). ECG revealed repolarization disorders (Fig. 2). Troponin and brain natriuretic peptide (BNP) were elevated. MRI revealed akinesia of the apex extending to the apical and middle segments of the adjacent LV walls with mildly reduced LV EF at 45%, along with akinesia of the inferior wall of the RV. LGE imaging identified transmural contrast in the apex and the apical and middle segments of the inferior, anterior, septal and lateral LV walls, with contrast in the inferior and lateral walls of the right ventricle (Fig. 3). Two possible diagnoses were evoked: necrotic sequela or ACM, given the presence of enhancement of both ventricles. To rule out the first diagnosis, the patient underwent coronary angiography which was normal. A genetic study was indicated but not conducted due to financial constraints. The diagnosis of biventricular ACM was retained based on Padua Criteria. Unfortunately, the clinical outcome was marked by sudden cardiac death (SCD) before the patient could benefit from an implantable cardioverted defibrillator.

## Discussion

ACM is a genetic heart disease characterized by progressive fibro-fatty myocardial replacement and arrhythmogenic features [3]. Initial Task Force criteria, revised in 2010, focused primarily on RV structural alterations and arrhythmogenic features to diagnose ACM [4]. However, there is growing evidence for LV involvement in ACM. Thus, the current classification of ACM includes 3 major phenotypes: (1) the original arrhythmogenic right ventricular cardiomyopathy (ARVC); (2) biventricular ACM; and (3) ACM with predominant LV involvement with no or minor RV abnormalities (ALVC) [5]. Diagnostic criteria for the left-sided phenotypic variants of ACM were only introduced in 2020 by an international expert consensus document, known as the “Padua criteria” [6], they are divided in six categories based on: (1) morpho-functional ventricular abnormalities, (2) structural myocardial tissue alterations, (3,4) ECG changes of ventricular depolarization and repolarization, (5) ventricular arrhythmias, and (6) familial/ genetic findings. The diagnosis of biventricular ACM is made if the patient meets a number of criteria from each phenotype, with the required presence of at least 1 criterion from the first or second category, either major or minor [6].

Using Padua criteria in our case, the presence of morpho-functional and structural myocardial abnormalities in both ventricles (diagnosed essentially by LGE CMR), as well as the presence of repolarization abnormalities on ECG, enabled the diagnosis. Hence, the presence of diagnostic criteria for the RV phenotype ensured us that the concomitant abnormalities of the LV were specific to ACM. Therefore, the diagnosis of biventricular ACM could be based on phenotypic features alone, without the need for genetic testing.

Adversely, an isolated LV phenotype of ACM can overlap with many differential diagnoses such as dilated cardiomyopathy, cardiac sarcoidosis, or more particularly myocarditis. Indeed, isolated LV LGE with subepicardial or mid-myocardial distribution could be mistaken for the consequence of a previous myocarditis, whereas histopathological studies are in favor of segmental ALVC. Moreover, myocarditis can induce ECG abnormalities similar to those described in ACM. Last, both myocarditis and ACM are responsible for non-ischemic myocardial scars that could generate ventricular arrythmias and SCD, especially in young people and athletes.

On one hand, this explains why nonischemic LV scars should not be trivialized and considered benign; instead, they should be subject to further investigation with the search of family history of cardiomyopathy, clinical history of acute myocarditis, the presence of symptoms and the arrhythmogenicity of the myocardial fibrosis. On another hand, this justifies the necessity of genetic testing to distinguish both pathologies. Indeed, in the presence of consistent LV phenotypic features, demonstration of a pathogenic or likely-pathogenic mutation of ACM related genes is mandatory for the diagnosis of ALVC [7].

Which raises the question whether or not our patient's first presentation of myocarditis was in fact an unrecognized isolated LV phenotype of ACM, followed by biventricular involvement leading to a definitive diagnosis of ACM a year later. Unfortunately, the clinical outcome was marked by SCD before the patient could benefit from an implantable cardioverted defibrillator.

## Conclusion

ACM is a genetic heart muscle disease that typically affects the RV. However, recent studies have discovered 2 other phenotypes involving either the LV alone or both ventricles. Therefore, physicians should always evoke the diagnosis of these newly-discovered phenotypes of ACM using Padua criteria whenever needed, and request genetic testing particularly if an isolated LV phenotype is suspected to confirm this diagnosis.

## Author contribution

Hajar El Ouartassi: Study concept, Data collection, Data analysis, Writing the paper. Raid Faraj: Study concept, Data analysis,Writing the paper. Zakariae Laraichi: Study concept, Data analysis, Writing the paper. Rhita Ezzahraoui: Data collection Zaineb Bourouhou: Data collection. Nawal Doghmi: Supervision and data validation. Mohamed Cherti: Supervision and data validation.

## Availability of data and materials

Data sharing is not applicable to this article as no datasets were generated or analyzed during the current study.

## Ethical approval

N/a

## Research registration

N/a

## Provenance and peer review

Not commissioned, externally peer-reviewed

## Patient consent

Written informed consent was obtained from the patient for publication of this case report and accompanying images. A copy of the written consent is available for review by the Editor-in-Chief of this journal on request.
